# Artificial Intelligence-Driven Discovery and Optimization of Antimicrobial Peptides Targeting ESKAPE Pathogens and Multidrug-Resistant Fungi

**DOI:** 10.3390/microorganisms14030591

**Published:** 2026-03-06

**Authors:** Calina Wu-Mo, Ariana Flores-González, Jezrael Meléndez-Delgado, Valerie Ortiz-Gómez, Héctor Meléndez-González, Rafael Maldonado-Hernández

**Affiliations:** 1 Department of Biology, University of Puerto Rico, Cayey Campus, Cayey 00737, Puerto Rico; calina.wu@upr.edu (C.W.-M.); ariana.flores@upr.edu (A.F.-G.); jezrael.melendez@upr.edu (J.M.-D.); 2 Department of Biology, School of Sciences & Technology, Ana G. Méndez University, Gurabo Campus, Gurabo 00777, Puerto Rico; ortizv3@uagm.edu; 3 Department of Internal Medicine, Ponce Health Science University, Ponce 00716, Puerto Rico; hjmelendez@viveclinicresearch.com

**Keywords:** artificial intelligence, antimicrobial peptides, ESKAPE pathogens, multidrug-resistant fungal pathogens, machine learning, deep learning, peptide design

## Abstract

Antimicrobial resistance (AMR) poses an escalating global health crisis driven by multidrug-resistant ESKAPE pathogens and emerging fungal threats such as *Candida auris* (*C. auris*). In response to this urgent need for new therapeutic strategies, antimicrobial peptides (AMPs) represent a mechanistically distinct alternative to conventional antibiotics due to their membrane-targeting mechanisms and a reduced propensity for resistance development; however, clinical translation has been hindered by toxicity, instability and manufacturing constraints. Recent advances in artificial intelligence (AI) are reshaping AMP discovery and optimization. Machine learning (ML), deep learning (DL) and transformer-based protein language models now enable improved prediction of antimicrobial activity, selectivity, protease stability and host toxicity. Generative approaches, including variational autoencoders, diffusion models and reinforcement learning, facilitate de novo multi-objective peptide design and pathogen-directed optimization against resistant bacteria and multidrug-resistant fungal pathogens. Integrated design–test–learn pipelines are accelerating iterative peptide engineering by tightly coupling computational prediction with experimental validation. Clinically used peptide-derived antibiotics such as polymyxins and daptomycin demonstrate the therapeutic feasibility of peptide-based antimicrobials, while investigational peptides, including pexiganan, illustrate ongoing translational progress. Although no fully AI-designed AMP has yet achieved regulatory approval, the accelerating convergence of computational modeling and experimental validation suggests a rapidly evolving translational landscape. Advancing scalable, surveillance-informed AI frameworks that integrate resistance data, predictive safety modeling and delivery optimization will be essential to accelerate the clinical translation of next-generation, multi-objective AMPs against high-risk resistant pathogens.

## 1. Introduction

AMR represents one of the most pressing global health challenges of the 21st century. Recent global estimates based on data up to 2019 indicate that AMR was associated with approximately 4.95 million deaths worldwide, including 1.27 million deaths directly attributable to bacterial resistance [1]. The rapid spread of multidrug-resistant pathogens has severely compromised the effectiveness of many conventional antibiotics, particularly in hospital settings [2]. Beyond its clinical impact, AMR is also closely linked to broader environmental pressures, including climate change, habitat disruption and biodiversity loss, creating reinforcing cycles that further increase human vulnerability to infectious diseases [3]. Consequently, combating AMR requires innovative antimicrobial strategies that are not only effective but also sustainable and scalable. Among the most critical resistant microorganisms are the ESKAPE pathogens (*Enterococcus faecium*, *Staphylococcus aureus*, *Klebsiella pneumoniae*, *Acinetobacter baumannii*, *Pseudomonas aeruginosa* and *Enterobacter* spp.), as well as emerging multidrug-resistant fungi such as *C. auris*, which together account for a substantial fraction of healthcare-associated infections worldwide [4]. These pathogens frequently exhibit resistance to multiple antibiotic classes and are often associated with biofilm formation, further complicating treatment and increasing morbidity, mortality and healthcare costs [5,6]. In this context, AMPs have gained attention as potential alternatives to many conventional antibiotics (e.g., β-lactams, fluoroquinolones and glycopeptides) due to their broad-spectrum activity and distinct mechanisms of action, such as membrane disruption through pore formation (e.g., barrel-stave and toroidal pore models) or the carpet model, which in many cases reduces the likelihood of resistance development compared to traditional small-molecule antibiotics [7,8]. In contrast to many conventional antibiotics that target specific intracellular enzymes or metabolic pathways, AMPs primarily act on microbial membranes, a mode of action that generally makes the emergence of resistance more difficult [9]. AMPs have shown activity against a wide range of bacterial and fungal pathogens, including multidrug-resistant strains and have been explored for applications ranging from systemic therapies to antimicrobial coatings for medical devices [7,10]. Nevertheless, despite their therapeutic potential, relatively few AMPs have achieved widespread clinical implementation for the treatment of infections caused by multidrug-resistant ESKAPE and fungal pathogens [11].

**Figure 1:**
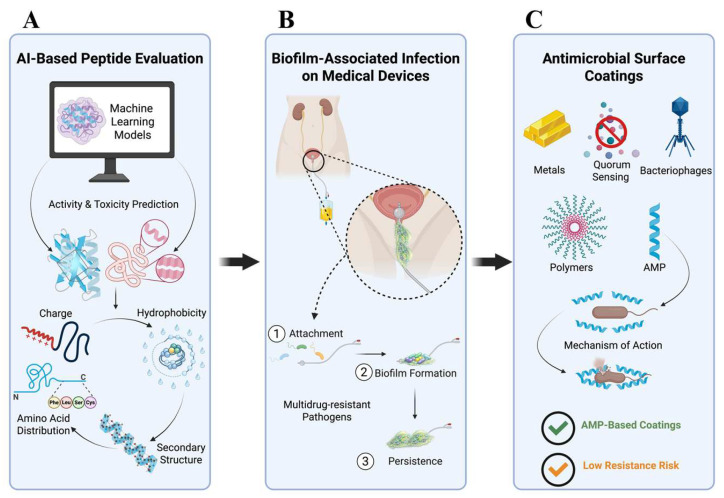
AI-guided antimicrobial peptide design and surface coating strategies to prevent biofilm-associated infections on medical devices. (**A**) Artificial intelligence models evaluate peptide properties, including charge, hydrophobicity, amino acid distribution, and secondary structure, to predict antimicrobial activity and toxicity. (**B**) Medical devices can become colonized by multidrug-resistant pathogens, leading to biofilm formation and persistence. (**C**) Antimicrobial surface coatings, particularly those based on AMPs, disrupt microbial membranes, reduce biofilm development and offer a lower risk of resistance compared to conventional antibiotics. Created in BioRender. Ortiz, V. (2026). https://BioRender.com/o2jl8eb.

A major limitation in the field is that AMP design and development face several critical challenges. Identifying peptides with true antimicrobial potential remains time-consuming, synthesis and optimization are costly, and many candidates exhibit unfavorable properties such as toxicity toward mammalian cells, poor serum stability, limited bioavailability, or susceptibility to proteolytic degradation [7,12]. In addition, achieving an optimal balance between antimicrobial potency, selectivity and safety remains a central bottleneck in translating AMPs from discovery to clinical use. These constraints highlight the need for more efficient and predictive frameworks capable of guiding peptide design toward clinically relevant property profiles. Recent advances in AI, particularly ML and DL, have begun to transform the landscape of AMP research by enabling data-driven discovery and rational optimization of peptide sequences [8,13,14]. ML-based models trained on large AMP databases can rapidly screen and prioritize candidate sequences based on physicochemical and structural features such as charge, hydrophobicity, secondary structure and amino acid distribution, thereby improving predictions of antimicrobial activity and toxicity while reducing the experimental burden (Figure 1A) [15]. More recently, generative models and structural prediction algorithms have further expanded these capabilities by enabling de novo peptide design and multi-objective optimization, integrating activity, selectivity and stability considerations into the design process [8,13]. These AI-driven approaches are particularly relevant for addressing healthcare-associated infections, which frequently arise from multidrug-resistant, biofilm-forming pathogens that colonize medical devices (Figure 1B).

Biofilm development significantly increases resistance to treatment and complicates clinical management [5,6]. Concurrent increases in life expectancy and expanding indications for joint arthroplasty have resulted in a substantial rise in prosthetic joint implantation worldwide, with a parallel increase in the incidence of periprosthetic joint infections (PJIs) [16]. PJI represents one of the most serious complications of orthopedic surgery and poses a significant clinical and public health challenge. The pathogenesis of these infections is largely driven by microbial biofilm formation on implant surfaces, which confers marked tolerance to antimicrobial therapy and host immune responses, thereby hindering eradication and frequently necessitating prolonged antimicrobial therapy and complex surgical interventions, including implant revision or removal [17,18,19]. In response, antimicrobial surface coatings based on metals, quorum-sensing (QS) inhibitors, AMPs, bacteriophages and enzymatic biofilm disruptors have been explored [7]. Among these strategies, AMP-based coatings have attracted growing interest due to their potent antimicrobial activity and reduced propensity for resistance development (Figure 1C). When immobilized on biomaterials, AMPs can prevent bacterial colonization, disrupt early biofilm formation and extend the functional lifespan of medical devices while minimizing patient complications [10]. Integrating AI-driven AMP design with surface engineering strategies, therefore, represents a promising avenue for mitigating the clinical burden of AMR. Together, these developments underscore the urgent need for a unified framework that integrates computational prediction, mechanistic understanding and translational validation to accelerate the development of next-generation antimicrobial peptides.

In this review, we examine how AI is reshaping AMP discovery and optimization, with particular emphasis on ML classifiers, DL architectures and generative peptide design frameworks. We provide a critical overview of current computational strategies for activity, selectivity, toxicity, stability and structure prediction, as well as pathogen-specific AMP design efforts targeting ESKAPE organisms and multidrug-resistant fungi. In addition, we discuss emerging delivery and formulation technologies, as well as the key experimental, clinical and manufacturing barriers that continue to limit peptide therapeutics. By integrating computational innovation with microbiological and biomedical perspectives, this review article aims not only to synthesize the current state of the field but also to outline future directions and translational opportunities for AI-driven AMP research.

## 2. Artificial Intelligence for AMP Discovery and Optimization

The rapid expansion of AMP sequence space, driven by genomic, metagenomic and synthetic peptide libraries, has created both an unprecedented opportunity and a major computational challenge for antimicrobial discovery [13,20,21]. Traditional experimental screening and rational design approaches, while powerful, are inherently limited by cost, time and the combinatorial explosion of possible peptide variants. Against this backdrop, AI, and particularly ML, has emerged as a transformative framework to accelerate AMP discovery and optimization by enabling data-driven exploration of sequence activity relationships at scale [22]. By learning patterns from curated AMP and non-AMP datasets, AI models can prioritize candidate peptides, predict biological activity and safety-related properties and guide iterative design cycles before experimental validation. Within the AI landscape, ML-based approaches have played a central role in bridging peptide biophysics and computational prediction [23]. These methods rely on the systematic transformation of peptide sequences into informative numerical representations, followed by the application of classification and regression models capable of capturing complex, nonlinear relationships between sequence features and antimicrobial function. Together, feature extraction strategies and ML classifiers form the backbone of modern in silico AMP screening pipelines, enabling not only the discrimination of AMPs from non-AMPs, but also the prediction of potency, selectivity, toxicity and other application-relevant properties.

### 2.1. Machine Learning for AMP Prediction

ML models for AMP prediction critically depend on the quality and biological relevance of extracted features from peptide sequences [8,13]. Because AMPs exert their activity through combinatorial interactions involving charge, amphipathicity, hydrophobic distribution and secondary structure, ML frameworks must capture both global physicochemical properties and local sequence motifs associated with antimicrobial function [24]. Feature extraction, therefore, serves as the foundation of ML-based AMP classification, enabling the transformation of raw peptide sequences into numerical representations suitable for computational learning.

#### 2.1.1. Feature Extraction: Physicochemical Descriptors and Sequence Patterns

Physicochemical descriptors commonly used in AMP prediction include net charge, hydrophobicity indices, hydrophobic moment, Boman index, aliphatic index, instability index and predicted isoelectric point [25]. These parameters reflect key determinants of microbial membrane interaction, such as the electrostatic attraction between cationic AMPs and anionic bacterial surfaces, or the hydrophobic forces that facilitate membrane insertion and disruption [25]. Additional structural descriptors such as predicted α-helical content, β-sheet propensity, amphipathic moment, and the propensity to form aggregates further refine activity prediction by capturing conformational features associated with peptide membrane dynamics [26,27]. Beyond global descriptors, ML models also leverage sequence-derived patterns including amino acid composition, k-mer frequencies, dipeptide and tripeptide motifs, pseudo-amino acid composition and autocorrelation features that encode spatial relationships along the peptide chain [26,28,29]. These representations allow algorithms to identify recurring antimicrobial signatures such as enrichment of lysine and arginine, clustering of hydrophobic residues and periodic distributions that favor amphipathic helices [4,30]. Collectively, these feature sets are commonly referred to as “handcrafted descriptors”, i.e., manually engineered features explicitly defined based on prior biochemical knowledge, including physicochemical properties, composition-based features and sequence-derived patterns. More advanced feature sets incorporate evolutionary information through position-specific scoring matrices, as well as sequence embeddings derived from protein language models, which capture higher-order contextual relationships not evident from handcrafted descriptors [31]. By integrating physicochemical and sequence-based features, ML algorithms gain the capacity to distinguish AMPs from non-AMPs, predict antimicrobial potency, estimate toxicity and identify key determinants of biofilm or fungal activity. The quality of feature extraction fundamentally determines model accuracy, generalization capacity and the reliability of downstream predictive tasks, making it a keystone of AI-driven AMP discovery.

#### 2.1.2. Classification Models

Classification algorithms represent the core of ML-based AMP prediction, enabling computational systems to distinguish AMPs from non-AMPs, predict antimicrobial potency and estimate properties such as hemolytic potential or biofilm activity [32]. Traditional supervised learning models, including Support Vector Machines (SVMs), Random Forests (RFs) and Extreme Gradient Boosting (XGBoost), have been widely adopted due to their robustness, interpretability and ability to handle heterogeneous feature spaces derived from physicochemical descriptors and sequence patterns [8,13,33,34,35]. SVMs are among the earliest and most influential classifiers used in AMP prediction [36,37]. By constructing optimal hyperplanes in high-dimensional feature space, SVMs maximize the margin between AMP and non-AMP classes, making them particularly effective for datasets with complex, nonlinear boundaries. Kernel functions such as radial basis function or polynomial kernels allow SVMs to model nonlinear relationships between peptide features and antimicrobial activity [38,39]. Many early AMP predictors, such as iAMPpred, AntiBP, and AMPScanner, relied heavily on SVMs due to their strong performance on small-to-medium-sized datasets and their resilience to overfitting [37,40,41,42]. RF classifiers operate by building ensembles of decision trees trained on bootstrapped subsets of the data, with each tree contributing a vote to the final prediction. RF’s ability to capture nonlinear feature interactions, handle large descriptor sets and provide feature-importance rankings has made it valuable for identifying the physicochemical determinants most associated with AMP activity [43]. In AMP discovery pipelines, RF models frequently outperform single-tree methods by reducing variance and improving generalization across diverse peptide families. XGBoost represents a more recent advancement in ensemble learning, leveraging gradient boosting with optimized tree-building algorithms to achieve high predictive accuracy. XGBoost excels at modeling complex, nonlinear interactions between features and often surpasses RF and SVM performance when trained on large, curated AMP databases [44]. Its strengths include regularization mechanisms that limit overfitting, the ability to integrate sparse or high-dimensional data and fast computational efficiency. In AMP prediction, XGBoost has been applied in multi-task or multi-output prediction settings, typically implemented through parallel or coupled models trained for related tasks such as antimicrobial activity, toxicity and peptide stability, thereby supporting multi-objective optimization [45]. Collectively, SVM, RF and XGBoost serve as foundational classification tools in AI-driven AMP discovery [46,47,48]. Their effectiveness depends on the quality of feature extraction and the diversity of training datasets, yet they continue to provide reliable predictive capabilities even as DL models gain prominence in the field. These classical ML approaches remain integral components of hybrid pipelines, often used as benchmarking models, interpretability tools, or pre-screening filters before more computationally intensive DL or generative models are applied.

## 3. Deep Learning Approaches

DL has become a transformative tool in AMP discovery by enabling models to learn complex patterns directly from peptide sequences without relying solely on handcrafted features [8,31,49]. Unlike classical ML methods, which depend heavily on predefined physicochemical descriptors, deep neural networks can infer nonlinear relationships, capture long-range dependencies and model higher-order biological interactions. These capabilities make DL particularly suited for sequence-based AMP prediction, potency estimation and toxicity classification. The most widely adopted architectures include Convolutional Neural Networks (CNNs), Recurrent Neural Networks (RNNs)/Long Short-Term Memory networks (LSTMs) and transformer-based peptide language models [50,51]. Hybrid frameworks additionally integrate structural and physicochemical information to enhance predictive accuracy.

### 3.1. CNNs, RNNs, LSTMs for Sequence-Based Prediction

CNNs have been widely applied in AMP prediction due to their ability to detect local sequence motifs and spatial patterns associated with antimicrobial activity [52,53]. By sliding filters across encoded peptide sequences, CNNs identify conserved motif clusters such as hydrophobic patches, cationic regions, or amphipathic periodicity that contribute to membrane interaction. CNN-based architectures have demonstrated high accuracy in classifying AMPs vs. non-AMPs and have been incorporated into several modern AMP prediction tools [8,13,52]. RNNs and particularly LSTMs excel at capturing sequential dependencies across peptide chains [20]. These models are designed to retain information over long sequence intervals, allowing them to learn order-sensitive patterns such as motif positioning, charge distribution periodicity and residue transitions relevant to membrane disruption or intracellular targeting. LSTMs have been applied to predict antimicrobial potency, hemolytic activity and even specific activity against Gram-negative vs. Gram-positive organisms [54,55,56,57]. In this context, the Reymond laboratory has made important contributions demonstrating the practical utility of sequence-based DL models for AMP design. The Reymond research group employed an RNN-based framework for the de novo design of AMPs, combining generative models with activity and hemolysis classifiers to identify novel, non-hemolytic antimicrobial peptides [58]. More recently, they performed a systematic comparison between classical ML models and large language models such as GPT-3.5 for the prediction of antimicrobial activity and hemolysis, showing that although LLMs exhibited promising potential, RNNs and SVMs trained on sequence- and structure-derived features provided more robust and consistent predictive performance [59]. These studies highlight the continued relevance of RNN-based architectures in AMP discovery while also offering a critical perspective on the current capabilities and limitations of emerging language models. Compared to classical ML models, CNNs and LSTMs allow the network to autonomously learn feature representations from raw sequences, reducing reliance on manual feature engineering and enabling improved generalization across diverse AMP families [60].

#### 3.1.1. Transformer Models and Peptide Language Models

Transformer architectures have emerged as one of the most impactful DL frameworks for peptide and protein analysis due to their capacity to capture long-range contextual relationships through self-attention mechanisms. Unlike recurrent models, transformers process entire sequences simultaneously, allowing them to learn global dependencies between amino acids with remarkable efficiency. When trained on large-scale protein sequence datasets, peptide-focused transformer models adapted from architectures such as ProtBERT, ProtT5 and ESM generate contextual embeddings that encode biochemical, evolutionary and structural information embedded within peptide sequences [61,62,63]. These embeddings often outperform handcrafted descriptors and classical ML descriptors, enabling improved prediction of antimicrobial activity, potency and toxicity [64]. In AMP discovery, transformer-based models have demonstrated strong performance in classifying AMPs from non-AMPs, estimating minimal inhibitory concentration (MIC) values and predicting hemolytic effects [65,66,67]. Their ability to model subtle patterns in residue arrangement also enhances predictions of antibiofilm, anti-Gram-negative and antifungal activity [68]. Furthermore, transformer language models serve as foundational components for generative design systems, which leverage learned peptide sequences to produce novel AMP candidates optimized for activity, stability, or therapeutic properties [8,13,69]. As a result, transformers represent a major advance in sequence-based AMP research and continue to redefine the scale and precision of peptide prediction.

#### 3.1.2. Hybrid Models Integrating Sequence and Structural Data

Hybrid DL models seek to improve predictive accuracy by combining information derived directly from peptide sequences with structural or physicochemical data [70,71]. This approach reflects the biological complexity underlying AMP function that antimicrobial activity emerges not only from amino acid composition but also from structural elements such as secondary structure, amphipathicity, solvent accessibility and conformational stability [4,7,72,73]. In these models, sequence encodings, whether derived from CNNs, LSTMs, or transformer embeddings, are integrated with structural descriptors obtained through computational tools such as AlphaFold, Rosetta, or secondary-structure prediction algorithms [74]. This integration creates a more comprehensive representation of each peptide, capturing both linear patterns and three-dimensional properties relevant to membrane interaction and intracellular targeting. Hybrid architectures have shown improved performance across several prediction tasks, including MIC estimation, membrane-binding affinity and toxicity classification [25,54,75]. They also enhance robustness when evaluating peptides that fall outside classical AMP motifs or exhibit unconventional amino acid distributions [13]. By incorporating structural information alongside learned sequence representations, hybrid models provide a more biologically grounded framework for AMP prediction and facilitate the rational identification of candidates with favorable therapeutic profiles. These advancements position hybrid DL systems as essential components of next-generation computational pipelines for peptide discovery and optimization.

## 4. Generative AI and Peptide Design

Generative AI has emerged as a transformative paradigm in AMP discovery innovations in the field of AMP discovery [13,21,76,77,78]. Unlike predictive models that classify or estimate the activity of existing sequences, generative models are capable of creating entirely new peptide sequences with optimized physicochemical and biological properties [8,13,21]. These architectures learn underlying statistical and functional patterns from large peptide datasets, enabling them to explore vast sequence spaces that are inaccessible to traditional empirical and rational design methods. Through controlled sampling, latent space manipulation and iterative optimization, generative AI has become a powerful tool for identifying novel AMPs with enhanced potency, reduced toxicity, improved stability and specific activity against multidrug-resistant (MDR) pathogens [8,21].

### 4.1. GANs, VAEs and Diffusion Models

**Figure 2:**
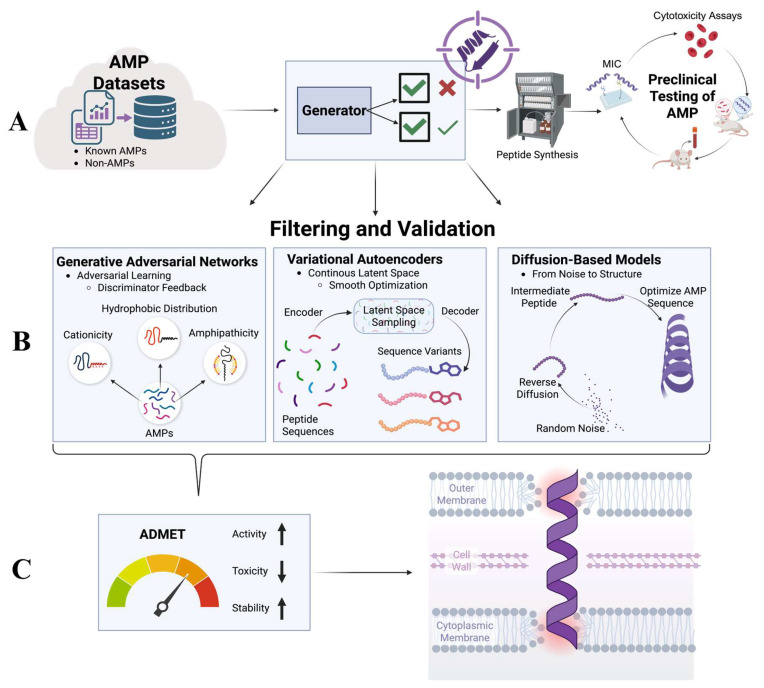
Generative  AI framework for de novo AMP design and optimization. This figure summarizes a generative AI-driven pipeline for AMP discovery, integrating conceptual, computational and functional stages. (**A**) Curated datasets of known AMPs and non-AMP sequences are used to train generative models that produce candidate peptides, which are subjected to in silico filtering and validation prior to experimental assessment and preclinical testing. (**B**) Three major families of generative architectures are illustrated. (**C**) Optimized AMP candidates are selected based on multi-parameter evaluation, including predicted antimicrobial activity, reduced toxicity, enhanced stability and are shown interacting with bacterial membranes, highlighting their translational potential for combating MDR pathogens. Created in BioRender. Ortiz, V. (2026). https://BioRender.com/qt1hlxh.

Generative Adversarial Networks (GANs), Variational Autoencoders (VAEs) and diffusion-based models represent three principal families of architectures used for de novo peptide generation (Figure 2) [79,80]. GANs operate through adversarial training in which a generator produces peptide sequences and a discriminator evaluates their resemblance to known AMPs [81]. This competitive learning process enables GANs to generate sequences that capture key biochemical patterns such as cationicity, amphipathicity and hydrophobic distribution. Early studies demonstrated that GAN-generated peptides could exhibit strong antimicrobial activity and maintain structural traits characteristic of natural AMPs [82,83]. VAEs take a different approach by encoding peptide sequences into a continuous latent space that represents underlying biochemical features [84,85]. By sampling and interpolating within this space, VAEs can efficiently explore sequence variants with gradually modified properties. This has proven especially useful for optimizing peptide length, adjusting hydrophobic moment and reducing predicted toxicity [86]. VAEs provide a smooth landscape for peptide design, enabling controlled modifications and multi-objective optimization [77]. Diffusion models represent the newest class of generative frameworks and have shown remarkable success in protein and peptide design [87,88]. These models iteratively transform random noise into structured peptide sequences through a learned reverse diffusion process. Because they are capable of generating highly diverse and structurally consistent sequences, diffusion models excel in producing AMPs that satisfy multiple design constraints simultaneously. Their ability to incorporate structural priors, activity predictors, or toxicity filters into the generation pipeline positions them as a leading methodology for next-generation AMP discovery [89].

#### Reinforcement Learning for Peptide Optimization

Reinforcement learning (RL) provides a complementary strategy for peptide design by guiding sequence generation according to predefined optimization objectives. In RL-based frameworks, peptides are generated through sequential decision-making processes in which an agent proposes amino acids one residue at a time. After each sequence is created, external models such as antimicrobial activity predictors, toxicity classifiers, or structural stability estimators serve as reward functions that evaluate the quality of the design. The agent then updates its policy to favor sequence patterns associated with higher rewards. This iterative optimization framework allows reinforcement learning systems to explore the sequence landscape more efficiently than random sampling or simple generative approaches. RL can incorporate multi-objective goals, promoting peptides that not only display predicted antimicrobial activity but also exhibit favorable absorption, distribution, metabolism, excretion and toxicity (ADMET) properties, reduced hemolysis, increased protease resistance, or targeted activity against specific pathogens or biofilm-associated phenotypes [13,90]. Recent advances have combined reinforcement learning with transformer embeddings and VAE latent spaces, resulting in hybrid frameworks capable of refining generative outputs toward clinically relevant peptide candidates [84,91,92]. RL thus plays a crucial role in transforming raw generative outputs into optimized AMPs tailored for therapeutic applications [93]. When integrated with predictive models and wet-lab validation, RL-based design pipelines significantly accelerate the development of novel peptides suitable for combating multidrug-resistant ESKAPE and fungal pathogens [8,94,95].

## 5. Structural Prediction and Validation

Structural characterization is essential for understanding AMP function, as biological activity is tightly linked to conformational dynamics, membrane interactions and the ability to adopt specific structural motifs such as α-helices, β-sheets, or extended amphipathic arrangements. AI has revolutionized structural prediction by enabling high-resolution modeling of peptide conformations and their interactions with microbial surfaces [7]. Modern tools such as AlphaFold and Rosetta have dramatically improved the accuracy of peptide structure prediction, while emerging AI-driven frameworks allow for the computational estimation of membrane binding affinity, insertion depth and interaction energetics [70]. Together, these approaches provide critical insights that facilitate rational peptide design, activity prediction and downstream experimental validation [96,97].

### 5.1. AlphaFold and Rosetta

AlphaFold, powered by DL and attention-based architectures, has significantly advanced the ability to predict protein and peptide structures from sequence alone [98]. Although AMPs are often shorter and more flexible than globular proteins, AlphaFold predictions are generated in an implicit aqueous environment and do not explicitly model membranes; however, the method can still provide useful estimates of secondary structure propensity, residue-level interactions and intrinsic folding tendencies that are relevant for membrane-active peptides [99]. These predictions help identify amphipathic helices, aggregation-prone regions, cationic clusters, or structural transitions associated with membrane disruption [100]. AlphaFold’s confidence metrics further allow researchers to determine which regions of a peptide are structurally stable or intrinsically disordered, both of which influence antimicrobial performance [101]. On the other hand, Rosetta remains a complementary tool with powerful capabilities for backbone sampling, side-chain optimization and energy-based refinement. Rosetta’s flexible peptide docking modules enable simulation of peptide interactions with lipid bilayers, protein receptors, or biofilm matrix components [102,103]. Its scoring functions provide quantitative estimates of stability, folding energy and structural compatibility, making it particularly useful for validating AlphaFold predictions or modeling noncanonical residues, cyclic peptides and post-translational modifications commonly found in engineered AMPs [104]. Together, AlphaFold and Rosetta establish a robust computational pipeline for structural modeling that guides rational design and experimental testing [105,106].

#### AI-Driven Binding and Membrane Interaction Predictions

Understanding AMP–membrane interactions is fundamental for predicting their antimicrobial activity and specificity. Emerging AI-driven tools now allow in silico simulation of peptide bilayer interactions, including estimation of membrane binding affinity, penetration depth, orientation and disruption potential [107]. DL models trained on molecular dynamics simulations and experimental datasets can infer whether a peptide is likely to embed within lipid bilayers, form pores, or destabilize membrane integrity [108]. These tools can also evaluate differential binding to Gram-positive vs. Gram-negative membranes, predict interactions with lipopolysaccharides and assess the likelihood of permeabilizing fungal cell walls enriched in ergosterol [60,109,110,111]. ML frameworks incorporating structural descriptors, hydrophobic moment and residue-level interaction energies have also been developed to estimate peptide selectivity and cytotoxicity. Such models help distinguish peptides that preferentially target microbial membranes from those that may damage mammalian cells [67]. Furthermore, hybrid approaches combining transformer-based embeddings with molecular interaction predictors enable high-throughput screening of thousands of candidate peptides prior to wet-lab validation [94]. AI-driven membrane interaction predictions thus serve as a critical bridge between computational design and experimental validation. By providing rapid, mechanistic insights into peptide membrane dynamics, these models significantly reduce the cost and uncertainty associated with AMP optimization and accelerate the discovery of candidates suitable for therapeutic development [7,112].

## 6. ADMET and Stability Prediction

Predicting toxicity, stability, and pharmacokinetic behavior is essential for advancing AMP from computational design to therapeutic application. While many peptides possess strong antimicrobial activity, their clinical translation is frequently hindered by hemolytic effects, rapid degradation by proteases, poor solubility, or aggregation tendencies that limit bioavailability [113]. AI has enabled systematic prediction of these liabilities by integrating physicochemical descriptors, structural features and DL representations derived from large peptide datasets. These computational tools allow researchers to screen candidate sequences for potential safety concerns, prioritize peptides with favorable ADMET profiles and design modifications that enhance therapeutic performance before entering experimental validation.

### 6.1. Hemolysis Predictors

Hemolysis prediction represents a critical step in AMP development due to the close relationship between membrane-disruptive antimicrobial mechanisms and potential damage to mammalian erythrocytes [114]. AI-driven hemolysis predictors rely on ML and DL models trained on experimentally validated hemolytic and non-hemolytic peptides. These models analyze charge distribution, hydrophobic moment, amphipathicity, sequence patterns and predicted secondary structure to identify features associated with erythrocyte lysis [115]. Transformer-based embeddings and graph neural networks have further improved predictive accuracy by capturing nuanced structural and contextual elements of peptide behavior [116]. By flagging sequences with high hemolytic potential early in the design pipeline, these tools reduce the likelihood of advancing toxic peptides and guide rational sequence modification to minimize off-target effects.

#### 6.1.1. Protease-Stability Models

Proteolytic degradation represents a major barrier to systemic AMP administration, as serum and tissue proteases rapidly cleave many peptide backbones [117,118]. AI-driven protease stability models help address this challenge by predicting cleavage sites and estimating overall peptide half-life in biological fluids [119]. These models incorporate sequence motifs, residue-level accessibility, secondary structure propensities and physicochemical descriptors to evaluate vulnerability to serine proteases, metalloproteases and other enzymatic classes [120]. DL approaches trained on large protease substrate datasets allow for accurate prediction of cleavage hot-spots and support the design of protective modifications such as D-amino acid substitution, cyclization, PEGylation, terminal capping, or incorporation of noncanonical residues [121,122,123]. By anticipating degradation pathways, protease-stability models enable proactive engineering of AMPs with improved pharmacokinetic profiles.

#### 6.1.2. Aggregation and Solubility Models

Peptide solubility and aggregation behavior strongly influence AMP efficacy, formulation, stability and manufacturability [124]. Peptides with excessive hydrophobicity, pronounced amphipathic clustering, or low sequence complexity may self-associate in solution, reducing bioavailability, altering membrane interactions and complicating large-scale production [125]. Aggregation can also compromise batch-to-batch reproducibility and limit achievable therapeutic concentrations, making solubility and aggregation propensity critical design constraints for peptide therapeutics [124,126]. AI-driven aggregation and solubility prediction tools provide valuable insight into these properties by analyzing sequence- and structure-derived features such as hydrophobic patches, charge balance, amino acid composition, structural flexibility and predicted solvent-accessible surface area [127]. ML models can identify aggregation-prone motifs and estimate the likelihood of peptide self-assembly under physiological or formulation-relevant conditions, enabling early-stage screening and prioritization of candidates with favorable developability profiles [128]. In parallel, regression-based and classification-based frameworks are increasingly used to predict solubility limits, aggregation thresholds and formulation compatibility, complementing activity and toxicity prediction pipelines [129]. More advanced approaches integrate structural information, including models derived from AlphaFold or Rosetta, with solubility and aggregation descriptors, capturing both linear sequence effects and three-dimensional conformational contributions to peptide behavior [130,131]. Such hybrid models allow the identification of buried hydrophobic surfaces, exposed aggregation hotspots, or conformational states that promote self-association. These predictions support rational sequence refinement, including residue substitutions, charge redistribution and modulation of amphipathicity, as well as formulation strategies such as PEGylation, cyclization, or carrier-based delivery systems that improve peptide stability and solubility under in vivo conditions [13]. Collectively, AI-guided aggregation and solubility models play a central role in de-risking AMP development by enabling the early elimination of poorly behaved candidates and guiding multi-parameter optimization toward peptides that balance antimicrobial potency with favorable physicochemical and pharmaceutical properties.

#### 6.1.3. AI-Guided Design of Amyloidogenic and Aggregation-Prone AMPs

While aggregation is often considered a liability in peptide therapeutics, increasing evidence indicates that amyloidogenic and aggregation-prone properties can also be exploited as a functional antimicrobial mechanism [132]. Several studies have shown that specific AMPs or peptide-like molecules can adopt amyloid-like conformations or induce aggregation of essential proteins in pathogenic microorganisms, thereby disrupting key cellular processes and inhibiting microbial growth [132,133,134,135]. Within this landscape, controlled aggregation should not be viewed merely as a formulation challenge but rather as a property that can be deliberately harnessed as a therapeutic strategy. On the other hand, recent studies, such as the exploration of amyloidogenic peptides derived from ribosomal S1 protein, demonstrate that AI-guided or computational modeling frameworks can be leveraged to design AMPs with targeted amyloidogenic or aggregation-prone properties, potentially disrupting essential bacterial proteins [133]. By learning sequence–structure aggregation relationships from experimental and structural datasets, ML and DL models can be trained to predict and optimize aggregation propensity in a context-dependent manner. Such approaches enable the balancing of solubility, stability and targeted aggregation behavior, facilitating the design of peptides that selectively promote aggregation of bacterial proteins while minimizing off-target self-assembly or host toxicity. Integrating aggregation-aware objectives into multi-parameter optimization pipelines, therefore, represents an emerging direction for AI-driven AMP engineering, extending current models beyond merely avoiding aggregation toward strategically exploiting amyloid-like mechanisms for antimicrobial activity.

## 7. Pathogen-Specific AMP Strategies: ESKAPE Organisms

AMR is a natural evolutionary adaptation in pathogens, which has been markedly accelerated by the misuse and overuse of antibiotics [136,137,138]. This trend has led to a steady increase in drug-resistant infections over recent decades, including strains resistant to last-resort therapies [139,140]. Among these, the ESKAPE pathogens represent the most clinically problematic group, frequently classified as MDR, extensively drug-resistant (XDR), or even pandrug-resistant (PDR) [139,141]. These organisms are responsible for a large proportion of nosocomial infections and cause severe complications in immunocompromised and immunocompetent patients, often increasing the risk of co-infections and mortality [137,140]. Their success as pathogens relies on a combination of structural and functional adaptations, including intrinsic resistance determinants encoded in their genomes and acquired resistance mechanisms arising from mutations and horizontal gene transfer [141,142,143]. While some resistance mechanisms are broadly conserved, others are species or strain-specific and directly impact the efficacy of therapeutic strategies. Unfortunately, the development of new antibiotics has not kept pace with the rapid acquisition and dissemination of resistance mechanisms [2,139,144]. Therefore, a detailed understanding of the pathogenic mechanisms and resistance strategies of ESKAPE organisms is essential for the rational development of novel therapeutic approaches, including pathogen-specific AMP-based interventions.

### 7.1. Mechanisms of Resistance

**Figure 3:**
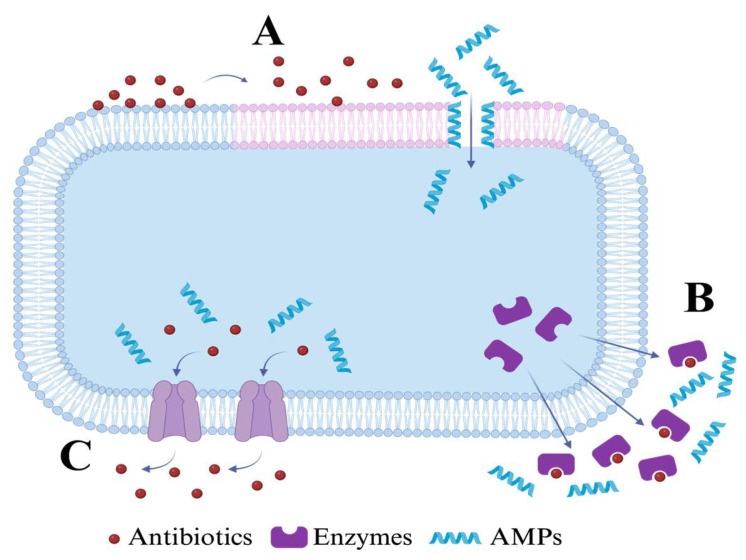
Antimicrobial peptide mechanisms of action and reduced susceptibility to classical antibiotic resistance. (**A**) After antibiotic exposure, the bacteria change wall components, lowering the affinity for the antibiotic. Despite this, some AMPs have the capacity to penetrate the membrane and kill the pathogen. Because AMPs target fundamental membrane properties rather than specific enzymatic or metabolic pathways, classical antibiotic resistance mechanisms such as target modification, (**B**) enzymatic degradation, or (**C**) efflux pump activation are largely ineffective, contributing to their activity against multidrug-resistant pathogens. Created in BioRender. Wu Mo, C. (2026). https://BioRender.com/pwzcmoo.

The general mechanism of action of AMPs involves binding to membrane components to disrupt membrane stability or synthesis and penetration of the cell to alter vital cell processes that ultimately lead to cell death [137,145,146,147,148]. Since it mostly relies on membrane interactions, the mechanisms of action are fewer and different from those of traditional antibiotics, which makes it more difficult for pathogens to develop resistance, as it lowers the mutation rate. The most studied mechanisms include Barrel-stave, Toroidal-pore and Carpet-model [148,149,150,151] (Figure 3). Though rare, some mechanisms of resistance have been found in different pathogens, especially ESKAPE pathogens [139,146,148,150]. These include surface alterations, efflux, target modification, phospholipid composition, stress responses of the membrane, enzyme production and drug targeting through mutation and horizontal transfer [139,146,148,150]. Concrete examples are resistance toward polymyxin from *Klebsiella pneumoniae* (*K. pneumoniae*), *Acinetobacter baumannii* (*A. baumannii*), *Pseudomonas aeruginosa* (*P. aeruginosa*), and *Staphylococcus aureus* (*S. aureus*) by modification of membrane composition for binding affinity reduction [139,150]. Another mechanism for resistance is the production and release of proteins and enzymes, where some are proteases from *Enterobacteriaceae* and *S. aureus*, elastases from *P. aeruginosa*, capsular polysaccharides from *K. pneumoniae*, extracellular proteoglycans from *P. aeruginosa* and *Enterococcus faecalis* (*E. faecalis*) [139,149,150]. Enterobacteriaceae can also create a shield for the cell surface that inhibits the penetration of drugs [150]. As pathogens possess fewer mechanisms of resistance against AMPs, this could result in an alternative to current available therapies.

Another mechanism of resistance against AMPs is biofilm formation, which is a common virulence factor from ESKAPE pathogens [139,148,149,152]. Biofilms are complex microbial communities surrounded by an extracellular polymeric matrix that contains exopolysaccharides, proteins and DNA [139,147,148,149,152,153]. This creates an environment persistence by persister cells, resistance against antimicrobial agents and helps evading host immune system [137,139,145,151,152,154]. Inside, microorganisms’ tolerance towards antibiotics and disinfectants increases, as penetration and eradication of the biofilms is nearly impossible with current available therapies [137,145,148,149,151,152,153,154]. Most ESKAPE pathogens have the capacity of creating biofilms, the principal ones being *P. aeruginosa*, *A. baumannii*, *S. aureus*, and *K. pneumoniae* in clinical settings [137,139,148,151,152,154]. Thanks to the difficulty of eradication, biofilms often lead to chronic infections with the possibility of surviving any kind of surface, including medical devices [137,149,153,154]. Some discovered AMPs have the capacity to inhibit biofilm formation and even destroy already established biofilms [137,146,147,148,149,150,151,153]. Biofilm formation represents a crucial factor for complications involving bacterial infections, as it remains difficult to eradicate while conferring resistance [7].

QS comprises molecular signals that modulate the expression of virulence factors [137,152,155]. In the case of biofilms, QS is important for biofilm formation and communication or coordination within the biofilm [137,148,151,152,155]. Virulence factors facilitate colonization and infection, starting disease, the evasion of the host’s first and second line of defense, creating cytotoxicity, damaged tissue, and inflammation [152,155,156]. These are expressed in molecules, substances or structures created by the pathogen, including pili, fimbria, and binding proteins [152,155,156]. *Enterococcus* ssp. have bacteriocins, aggregation factors, and determinants for drug resistance; specifically, *Enterococcus faecium* (*E. faecium*) expresses proteins for adhesion and colonization, enzymes, cytotoxicity and biofilm formation [155,156]. In the case of *S. aureus*, there are also proteins for adhesion and colonization, cytotoxicity, evasion of the host immune system and biofilm formation as virulence factors [155,156,157]. For *K. pneumoniae*, there is expression of pili, fimbriae, QS autoinducers, aggregative adhesion, capsular polysaccharides, lipopolysaccharide, iron uptake, and biofilm [152,155,156]. In *A. baumannii*, there is protein synthesis, phospholipases, capsular polysaccharides, lipopolysaccharides, membrane and QS proteins, metal absorption, cytokines and biofilms [152,156]. *P. aeruginosa* presents virulent molecules, enzymes, peptides, flagella, fimbria, lipopolysaccharide, pili, effector protein, cytotoxicity, phospholipases, secondary metabolites, polysaccharide, exopolysaccharide and biofilms [2,155,156]. However, the mechanisms of virulence in *Enterobacter* spp. have yet to be studied thoroughly [156]. The inhibition of virulence factors may become a potential target for therapeutic drugs to limit infection and biofilm formation.

#### Preclinical and Clinical Evidence

Several antimicrobial peptides and peptide-derived antibiotics have progressed into clinical use, particularly for the treatment of multidrug-resistant infections. Notable examples include polymyxins and daptomycin, widely employed against Gram-negative and Gram-positive pathogens, respectively. In addition, peptide-based compounds such as ramoplanin, omiganan and nisin-derived molecules have been evaluated in clinical settings for their broad-spectrum antimicrobial properties [158,159,160,161]. Pexiganan, a synthetic analog originally derived from an antimicrobial peptide isolated from the skin of the African frog *Xenopus laevis*, has demonstrated broad-spectrum activity against both Gram-negative and Gram-positive bacteria. Mechanistically, pexiganan disrupts bacterial membranes through toroidal pore formation, leading to membrane destabilization and oxidative stress [162]. Its efficacy is particularly notable against resistant strains. However, like many AMPs, its activity may be compromised under physiological conditions due to degradation or reduced stability. To overcome these limitations, recent studies have explored nanoformulation strategies, including combinations with chitin, cholesterol, sodium alginate and especially chitosan nanoparticles. The poly-β(1-4)-N-acetyl-D-glucosamine structure of chitosan enables specific interactions with biological molecules and enhances applications in controlled drug delivery, tissue engineering and wound healing. Furthermore, covalent coupling approaches such as EDC/NHS chemistry have been employed to improve structural stability and functional performance, particularly in biofilm-associated infections and device-related contexts [163,164]. Other peptide therapeutics, including gramicidin derivatives and host-defense peptide analogs such as hLF1-11, have demonstrated antibacterial and antifungal potential in preclinical and clinical studies. Glycopeptide antibiotics such as teicoplanin, dalbavancin, oritavancin, and telavancin are also used against resistant Gram-positive infections, although they are often classified as peptide-derived antibiotics rather than classical antimicrobial peptides. Furthermore, emerging agents such as murepavadin, which targets lipopolysaccharide biosynthesis in Gram-negative bacteria, highlight the continued interest in peptide-inspired antimicrobial strategies [158,159,161]. Collectively, these examples illustrate the translational potential of peptide-based antimicrobials, although many AMP candidates remain under investigation to address challenges related to stability, toxicity and large-scale clinical implementation.

## 8. AI-Designed AMPs Against Multidrug-Resistant Fungal Pathogens

The current limitations of antifungal therapies have exposed a critical gap in available treatment options for invasive fungal infections. Recent global estimates indicate that fungal diseases affect billions of people annually and are associated with several million deaths worldwide, underscoring their substantial and often underappreciated impact on public health [165]. Among these infections, candidemia remains one of the most frequent causes of bloodstream infections in intensive care units, with reported mortality rates exceeding 30%, which may be even higher in patients with septic shock or severe comorbidities [166]. In parallel, the emergence of *C. auris* as a rapidly spreading multidrug-resistant fungal pathogen has intensified concerns regarding the limited efficacy of current antifungal drug classes and the growing burden of untreatable or difficult-to-treat infections [69,167]. Against this backdrop, AI and ML approaches are increasingly being applied to accelerate the discovery and optimization of AMP, including antifungal peptides (AFPs) [165,168,169]. By enabling data-driven exploration of sequence-activity relationships and multi-parameter optimization, AI-guided frameworks offer a promising strategy to identify novel peptide candidates with improved efficacy and safety profiles.

### 8.1. Why Candida auris Belongs with ESKAPE-Level Threats

Antifungal resistance is increasingly recognized as a major, yet often overlooked, dimension of global microbial threats. Among emerging fungal pathogens, *C. auris* has become the most concerning example of a multidrug-resistant yeast. This aggressive organism exhibits unique defense and survival mechanisms that complicate treatment, infection control and environmental eradication. First identified in Japan in 2009, *C. auris* rapidly emerged as a significant public health threat [170]. In 2022, the World Health Organization classified *C. auris* as a critical-priority fungal pathogen. Globally, fungal infections affect approximately 6.5 billion people annually, contributing to an estimated 3.7 million deaths each year [165]. Reflecting its clinical severity, the U.S. Centers for Disease Control and Prevention has designated *C. auris* as an urgent health threat due to its multidrug resistance, persistence in healthcare environments and associated high mortality rates. Mortality estimates vary depending on comorbidities and outbreak settings, with reported rates ranging from 27.5% to 36.1% in certain cohorts [171]. In the United States, reported cases increased dramatically from 479 in 2019 to 1471 in 2021, with a sharp surge between 2020 and 2021, during which cases nearly tripled, reaching a cumulative total of 4401 [172].

Transmission occurs primarily in healthcare settings, including hospitals, long-term acute care facilities and nursing homes, particularly among patients requiring ventilatory support [172]. Although the precise origins of *C. auris* remain under investigation, it has been hypothesized that environmental pressures, possibly in wetland ecosystems, contributed to its emergence as a human pathogen [170,172]. Importantly, *C. auris* produces extracellular polymeric substances that promote surface adhesion, biofilm formation and resistance to antifungal agents. These properties facilitate persistence on medical devices such as catheters, implants and prosthetics, as well as prolonged survival in clinical environments [7]. Given these characteristics, *C. auris* exhibits epidemiological, resistance and environmental persistence features comparable to ESKAPE-level bacterial threats. Addressing this growing challenge requires the development of innovative therapeutic strategies, including AI- and ML-guided design of targeted antimicrobial peptides.

#### 8.1.1. AMP Mechanisms Against Fungal Pathogens

Given the limitations of current antifungal options, there is an urgent need to develop alternative therapeutic strategies. AI and ML have recently emerged as transformative tools for the discovery and optimization of AMPs, short, with rapid, broad-spectrum activity and reduced potential for resistance development [69,165,168]. AFPs are a specified subgroup under AMPs that have selective activity against pathogenic fungi [169]. They are defined as catatonic molecules with an affinity for cell membranes, where the activity is controlled by distinct properties of the cell (e.g., hydrophobic residues, chain length and the amphiphilic nature of the sequence) [169]. Despite the lack of studies surrounding AFPs, a few have made it into late-stage clinical trials, showing positive results against *Candida* spp. and *Aspergillus* spp., which are mainly responsible for invasive fungal human infections [169,173]. With fungal infections increasing and the rise in *C. auris* as a new multidrug-resistant pathogen, public health needs novel treatments. For example, recent research has demonstrated that ML classifiers can efficiently discover and classify drug-resistant mutations in *C. auris* [165]. Emerging mutations such as R278H in the ERG10 gene, I466M and Y501H in the ERG11 gene, may influence fluconazole, amphotericin B and micafungin resistance [165].

Nonetheless, studies such as Wang Y et al. are training AI/ML models to sequence AMPs with adequate novelty and diversity. In their research, they found 25 out of 40 peptides that exhibited antimicrobial and antifungal activities. Among the 25 AFPs/AMPs, five had selective activity against fungal species, while three indicated specificities against some bacterial species [168]. This indicates that AMPs are specific to cell wall or membrane components, depending on the fungal or bacterial species. In their study, Sousa et al. reference the use of bioactive peptides, which are derived from different natural sources involving insects, plants and marine organisms, with potential antifungal properties. Findings showed that an AFP named Blap-6 was the standout prospect to employ against *Candida* spp., demonstrating capacity against biofilm inhibition [174]. However, Marciano, CL et al. mention that over 5000 AMPs have been characterized, but only 31 have achieved pre-clinical trials, 38 are in phase I–III, and 17 are on the market after being FDA-approved [173]. The scarcity of AMPs and AFPs on the market is due to the challenges presented in the middle of the procedures, for instance, pharmacokinetics and the regulation of peptide use in vivo (e.g., selective toxicity, low bioavailability, proteolytic degradation and a lack of standardization in establishing specific legislative guidelines) [173]. Despite these difficulties, AMPs and AFPs are a new promising strategy to fight against the rise in AMR and antifungal drug resistance, but further research is required.

#### 8.1.2. Membrane Disruption

The plasma membrane plays a crucial role in AMPs and AFPs targeting. It is essential for maintaining cellular integrity, regulating molecular traffic and mediating interactions with the environment [175]. From a biochemical perspective, the plasma membrane controls fluidity, permeability and possesses the ability to respond to stress (e.g., oxidative, osmotic and drug-induced). Differences in AMP selectivity between fungal and bacterial cells to precise AMPs and AFPs have to do with the amount of ergosterol levels, which can affect fungal virulence. AFPs can be optimized to target ergosterol while showing low cytotoxicity in mammal cells in comparison with other antifungal drugs [176]. Moreover, environmental components can influence membrane–peptide interactions in fungi cells. To further explain the differences, bacterial membranes consist of a high content of anionic phospholipid and strong electrostatic attraction to cationic AMPs, which facilitates peptide binding, incorporation into the lipid bilayer, membrane disorganization, pore formation and rapid cell rupture [177]. In contrast, fungal membranes contain ergosterol (the main fungal sterol) and glycosphingolipids, more specifically glucosylceramide (GlcCer), which are both surrounded by a solid cell wall composed of chitin and β-glucans [169]. These features influence key aspects of fungal cell susceptibility, such as the reduction in electrostatic interactions, requirement of more specialized peptide recognition and contribution to selective AFPs. Furthermore, numerous AFPs not only eradicate fungi solely by membrane lysis but also cause oxidative stress, mitochondrial dysfunction, programmed cell death and autophagy [111]. Likewise, environmental factors may contribute to the interactions between peptides and the fungal membrane. Two-valent cations, such as Ca^2^^+^ and Mg^2+^, can improve antifungal activities while attenuating antimicrobial potency, depending on peptide structure and ionic strength [169].

For example, cathelicidin LL-37 is the most explored AMP due to its potential for clinical application as an antibacterial, antibiofilm, antiviral, antifungal, immunomodulatory and tumor repressor peptide [178]. It has been demonstrated to work against various multidrug-resistant bacteria, yet only a few have explored its antifungal properties [179]. Rather et al. demonstrated that human cathelicidin LL-37 exhibits antifungal activity against *C. auris* clinical isolate through a membrane disruption mechanism. LL-37 affected the pathogenic fungal plasma membrane, as findings proved by ATP and protein efflux, propidium iodide uptake and amplified lipid peroxidation, pointing to loss of membrane activity. Permeabilization of the membrane triggers oxidative stress, defined by elevated reactive oxygen species, dysregulation of antioxidant enzymes and accumulation of lipid peroxidation products, further affecting membrane operability [180]. Moreover, these outcomes resulted in mitochondrial dysfunction, inhibition of DNA synthesis and the inhibition of the fungal cell cycle at the S phase [181]. Collectively, the findings corroborate a framework where LL-37 primarily targets *C. auris* plasma membrane, causing permeability and oxidative stress that interactively catalyzes fungicidal activity, with a low chance of developing resistance.

## 9. Delivery Systems and Formulation Strategies for AMPs

The powerful antimicrobial activity of AMPs has developed as a potential solution to the rising AMR. However, the clinical translation of AMPs has presented numerous challenges due to in vivo bioavailability and biosafety concerns, including cytotoxicity, susceptibility to protease degradation and short half-life [182,183,184]. Therefore, the development of an efficient delivery system for AMPs as a therapeutic treatment is necessary to overcome these barriers and AMR. The usage of AI to perform formulation strategies to enhance the delivery of AMPs has emerged as a possible solution for the biological limitations of AMPs, as it has been proven that formulation strategies using nanoparticles (NPs), PEGylation, cyclization, and D-amino acid substitutes have enhanced stability, protease degradation resistance, solubility and circulation time [185,186,187].

### 9.1. Encapsulation Strategies for Drug Delivery

**Figure 4:**
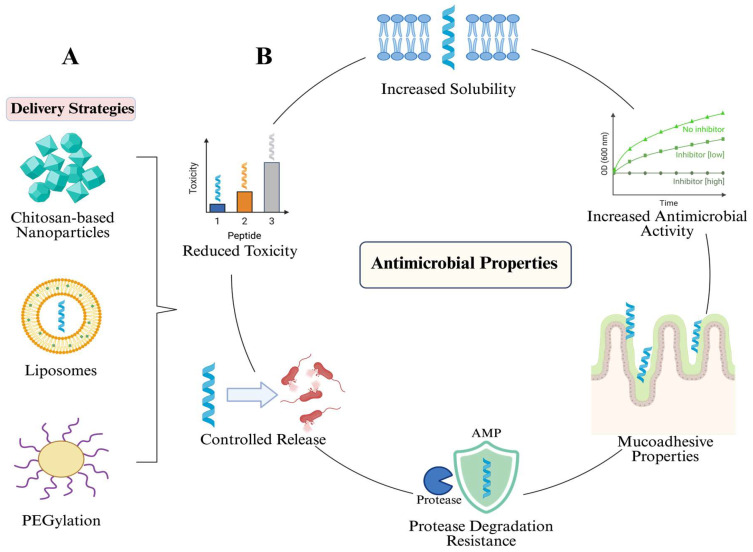
Delivery strategies for AI-designed antimicrobial peptides. Schematic representation of formulation strategies used to enhance the stability, bioavailability, and antimicrobial performance of AI-designed AMPs. (**A**) Overview of complementary delivery approaches, including chitosan-based, liposomal encapsulation and PEGylation. (**B**) These strategies protect AMPs from proteolytic degradation, reduce aggregation and toxicity, prolong circulation time, improve peptide stability, antimicrobial activity and biosafety through the use of mucoadhesive and biocompatible carriers. Created in BioRender. Melendez, J. (2026). https://BioRender.com/67llqna.

Research on drug delivery systems for AMPs is gaining increasing attention, as the distinctive features of NPs, including their nanometer-scale size, intrinsic antimicrobial properties, tunable physicochemical surface characteristics, ease of application and compatibility with formulation strategies have positioned them as promising candidates to improve the pharmacokinetic (PK) profiles of AMPs, as previously demonstrated for other small-molecule therapeutics [182,188]. Encapsulation approaches designed to improve stability and drug delivery are generally classified into four principal categories: inorganic (e.g., titanium oxide), polymeric (e.g., chitosan and polyethylene glycol (PEG)), lipid-based (e.g., liposomes) and other structured systems (e.g., dendrimers, carbon nanodots, and quantum dots) (Figure 4A) [182,189,190]. Chitosan, a natural biopolymer derived from chitin deacetylation chemically composed of β (1,4)-linked units of glucosamine units (2-amino-2-deoxy-β-d-glucopyranose) and segments of N-acetylglucosamine units (2-acetamino-2-deoxy-β-d-glucopyranose). The increase in studies regarding chitosan-based nanoparticles (CSNPs) in AMPs has gained attention due to their effectiveness in enhancing their biocompatibility, biodegradability, antimicrobial properties and biosafety caused by their natural glycosaminoglycan structural affinity [191,192,193,194]. The mucoadhesive properties of chitosan contribute significantly to its bioavailability and biocompatibility, as it represents a viable approach to drug delivery [195] (Figure 4B).

On the other hand, PEGylation, a covalent modification using the synthetic polymer PEG, has been proven to overcome biopharmaceutical limitations. PEGylation addresses limitations in AMPs by improving half-life, stability, solubility, safety and protease degradation resistance, while retaining its antimicrobial activity [196,197,198,199,200]. The utilization of PEGs and their effectiveness in protein molecules, such as AMPs, has a direct correlation with their molecular weight. As molecular weight increases, the half-lives, retention in the body and resistance to protease degradation increase, requiring a more complex metabolic process in the human body to excrete. Instead, when molecular weight decreases, PEGs lose their efficacy as they are easily excreted in urine [201]. Moreover, PEGylation can improve biocompatibility and biosafety properties of AMPs by preventing aggregation and improving solubility [199,202]. PEGylation has been implemented in CSNPs to improve stability, hydrophilicity and reduce toxicity. However, PEG chains length must be carefully optimized is needed to be taken into consideration as longer chains limit NPs’ cellular intake properties [203].

Liposomes, composed of lipid bilayers, are among the most widely used nanoparticles for drug delivery systems via encapsulation. They are considered one of the most promising nanoparticles for AMP delivery systems, as they are currently being used in medical treatment for their efficiency in vivo [204]. The biocompatibility advantages of liposomes can be altered by the adjustment of lipid composition due to their ability to attach to hydrophilic and hydrophobic molecules. Moreover, biocompatibility and biosafety properties are demonstrated through the reduction in toxicity and the improvement of release and encapsulation efficiency, as well as antimicrobial properties [185,205,206]. A unique property that liposomes exhibit for proper AMP encapsulation is their in vivo distribution due to the efficient tissue penetration through absorption or endocytosis mechanisms [185,207,208,209]. Liposomes also address one of the most crucial limitations of AMPs at present, immunogenicity, which has been proven to reduce protease degradation via encapsulating AMPs. However, a concern in the incorporation of liposomes in cationic AMPs has emerged, as leakage of these antimicrobials can occur due to hydrophobic and electrostatic interactions [210,211]. The high production cost of liposomes presents a limitation to the mass application and production for use in the biomedical field as the AMR pandemic rises. Still, the use of AI has emerged as a possible solution for cost reduction [212].

#### 9.1.1. Cyclization and D-Amino Acid Modification

Cyclization is a chemical synthetic strategy used to improve the PK profiles of AMPs. One of the most used strategies is macrocyclization, which can be achieved by the condensation of their amino and carboxyl group terminals [213,214]. One of the most widely used cyclization techniques is head-to-tail cyclization using the beta-lactam moiety as a bridge between the N- and C-terminals [215]. The cyclization of AMPs enhances their stability, as the absence of amine and carboxylic acid groups decreases the metabolic effects of exopeptidases [216]. Therefore, cyclization brings a possible solution to the protease degradation limitation of AMPs, an important limitation to overcome due to the inactivity of some AMPs during MIC procedures. Moreover, the cyclization of AMPs can counteract AMP limitations by decreasing toxicity and increasing antimicrobial activity [217]. Enhanced antimicrobial activity is acquired due to the improved penetration and membrane permeability, which leads to bacterial death and membrane disruption [110].

D-amino acids (DAAs) are enantiomers of naturally occurring L-amino acids and are present in numerous neurobiological and endocrine processes and functions in mammals and humans, such as learning, behavior, neuroprotector for neurodegenerative disorders when the NMDA receptor is mutated and the synthesis of hormones [218]. Peptides composed of natural L-amino acids, particularly those enriched in residues such as arginine and lysine, are highly susceptible to proteolytic degradation by trypsin and other plasma proteases, which has motivated the incorporation of DAAs as a strategy to improve peptide stability. Moreover, substitution with DAAs may enhance other properties, such as antimicrobial activity, by increasing permeability into bacterial membranes and eukaryotic tissues [122,219]. However, the excessive modifications of LAAs with DAAs in AMPs may provoke a setback to their advantages by presenting toxicity, immunogenicity and other biosafety in vivo concerns [187,220]. Although chemical synthesis strategies have been proven on DAA peptides to reduce production cost, the need for raw materials to produce DAAs is still considered a drawback [221].

#### 9.1.2. AI for Formulation Optimization

The incorporation of AI poses a unique approach to the discovery and design of AMPs. The primary objective of AI-driven formulation strategies is to counteract the time spent on the discovery and design experiments of AMPs to address rapidly growing AMR. Moreover, it is a promising tool for the modification of AMPs to overcome biosafety and bioavailability concerns previously mentioned with the use of AI technologies such as ML and DL [47,222]. The addition of AI technologies into the discovery and prediction of AMP sequences with ML and DL use can be separated into different levels: (1) classification of sequences as AMP or non-AMP, (2) bioactivity differentiation, (3) taxonomy selectivity, (4) AMP antibacterial activities and (5) the possibility of predicting MIC results in specific bacteria [69]. To achieve a precise discovery and prediction of AMPs, the rise in specialized computational databases is utilized. The most widely used databases to advance the discovery and design of AMPs are the Antimicrobial Peptide Database (ADP), the Collection of Anti-Microbial Peptides (CAMP) and the Database of Anuran Defense Peptides (DADP), which can help in the development of Level 5. Databases such as the Data Repository for Antimicrobial Peptides (DRAMP) and Database of Antimicrobial Activity and Structure of Peptides (DBAASP) can overcome the clinical translation problems present in AMPs by predicting toxicity and potential clinical peptides with the prior clinical trial peptides in the database. However, some ML programs show notable issues, such as the Deep-AMPep30, which has a limit of 30 amino acids for AMPs prediction sequences. Moreover, DBAASP is one of the few programs that can predict Level 4 and Level 5 for the discovery and design of AMP sequences against specific pathogens [223,224]. As multiple peptides are present in these databases, the need for a program for wet-lab research experiments, such as quantitative structure-activity relationship (QSAR), ensures that AMP bias is discarded by comparing their antimicrobial activity and structure [76,225,226]. Another type of AI tool for the discovery and design of potentially clinical AMPs is deep neural networks, which are used to predict the antimicrobial activity against specific pathogens and hemolytic activities. The use of these AI tools has improved AMPs’ limitations, such as stability, immunogenicity and protease degradation, and has given AMPs’ flexibility properties to improve Level 5 in terms of the implementation of AMPs into wet-lab and clinical settings. Residues such as tryptophan, tyrosine and phenylalanine can be incorporated into AMPs to improve peptide-membrane interactions for cell permeability and antimicrobial activity [215,227]. One of the latest AI tools for the improvement of Level 5 is AMPGen, which has the ability to generate AMPs not present in the databases mentioned before, while having high antimicrobial activity. AMPGen uses an order-agnostic autoregressive diffusion model generator pre-trained by the UniClust30 database, creating a new dataset called AMP-MSA. Moreover, an XGBoost-based discriminator and a Long Short-Term Memory (LSTM) scorer are used. The XGBoost-based discriminator is used for the determination of AMPs and non-AMPs through the physicochemical properties of the sequences generated, and the LSTM scorer is used for the recognition of AMP properties and effectiveness in specific pathogens. The combination of these three tools in the AMPGen is a revolutionary tool, as many AI tools do not cover the five levels of AMP discovery and design [69,228].

#### 9.1.3. Challenges for Systemic vs Topical Delivery

Regardless of the effectiveness of AMPs against MDR pathogens, systemic and topical delivery challenges remain in clinical translation. The delivery of medication can be done via an oral, intravenous (IV), intramuscular, subcutaneous, topical or inhalation route. The administration route used and the medication’s physicochemical factors present in AMPs, such as lipid solubility and ionization degree, determine the absorption effectiveness. The oral consumption of AMPs might face systemic delivery challenges due to poor absorption and permeability in the gastrointestinal system. The presence of digestive enzymes presents a threat to AMPs as they have been proven to be rapidly degraded when reaching the gastrointestinal region, reducing their half-life. IV administration of AMPs presents similar challenges as numerous proteases in the bloodstream, kidneys and liver also reduce their in vivo half-life. On the other hand, protease degradation is also a challenge in topical delivery, as skin proteases can fragment AMPs. As previously mentioned, pH is a key factor for AMP effectiveness; therefore, the drastic pH changes in wounds present adversity to the potential biomedical properties of AMPs in the human body [200,229,230,231,232,233]. Thus, the challenges mentioned emphasize the necessity of new delivery systems and formulation strategies moving towards clinical translation.

## 10. Manufacturing Challenges

Although AMPs have emerged as a promising solution to combat AMR, their manufacturing complexity remains a major translational bottleneck, limiting both economic feasibility and scalability [118]. In practice, production is often restricted to small-scale laboratory synthesis, as costs increase substantially with peptide length, purity requirements and chemical modifications [160,183]. Moreover, the complexity and sensitivity of the human immune system necessitate the development of strategies to improve bioavailability, as AMPs can lose efficacy under certain physicochemical conditions and may induce cytotoxic effects. Consequently, additional modifications (e.g., cyclization, DAAs) are often required, making the screening process more time-consuming and the manufacturing process more costly and complex [234,235]. However, the emergence of AI-driven tools has enabled a paradigm shift by bridging laboratory and clinical manufacturing, thereby improving the prospects for clinical translation [236]. Moving forward, the development of new technologies and strategies to streamline peptide synthesis will be crucial to achieving scalable industrial production of AMPs.

### 10.1. AI for Cost Reduction and Manufacturability Predictions

The screening and purification processes make the discovery and design of bioefficient and therapeutic AMPs time-consuming and costly. The emerging AI machinery is currently being developed to counteract the time-consuming and expensive limitations of the standard screening processes. Current AI/ ML tools have been proven to significantly lower the cost of production and improve the efficiency of the manufacturing process of AMPs [237]. AI-driven sequence generation platforms, such as ProGen and ProtGPT2, have further accelerated the identification of peptide candidates with improved manufacturability and predicted biological activity. Moreover, these models enable the exploration of sequence diversity while reducing the need for extensive experimental screening. Moreover, current AI tools can predict and optimize the shape and energy of the binding interaction between the AMP protein and its target protein based on data such as 3D structure and functional site. Examples of AI platforms with these capabilities include AlphaFold by DeepMind and Rosetta Fold by the University of Washington. Although real experimentation processes are proven to be the most direct way to validate the bioefficacy and biosafety parameters of AMPs, they also result in the costliest path. The use of AI addresses one of the major limitations AMPs exhibit, toxicity, with two of the most widely used databases: ConoServer and ArachnoServer. ToxinPred, an AI/ML algorithm capable of sequence amino acid composition, dipeptide composition and binary profile, is widely used in the AMP toxicity prediction, as it integrates motif-based toxicity region predictions [238,239]. A more specific type of toxicity, hemolysis, is also addressed with AI predictive models, such as HemoPI and HemoPred, for the prediction of hemolytic activity of AMPs, a clinical translation limitation involving the lysis of red blood cells [240,241]. As mentioned before, DNNs such as AMPDeep, with the assistance of ProtTrans, a pre-trained protein representation program, have the capability of predicting the hemolytic activity of AMPs when it is fine-tuned on a small-scale AMP dataset [242]. Most AI models achieve high accuracy on predicting hemolytic and non-hemolytic AMPs; however, they also have a limitation on predicting high vs low hemolytic AMPs [238]. Although these AI models and tools focus mainly on the biological effects of AMPs, the prediction capabilities these machines have correlate directly with the cost reduction in the manufacturability.

#### Regulatory Considerations for Peptide Therapeutics

Despite the antimicrobial activity of AMPs providing a solution to AMR, they still face challenges in clinical translation. The major concern in AMP clinical translation is the hemolytic and cytotoxic activities they may exert on patients at antimicrobial concentrations. To address these toxicity issues, regulatory and preclinical evaluations are required. Pharmacological and toxicological parameters such as EC50 and LD50 are commonly used to assess efficacy and safety, wherein EC50 refers to the concentration producing 50% of the maximal biological effect, and LD50 refers to the dose that causes lethality in 50% of test organisms in vivo [243]. These metrics, together with additional in vitro and in vivo assays, provide critical information on therapeutic windows and safety margins. Moreover, cationic AMPs have been shown to lose antimicrobial activity in clinical saline settings due to their loss of electrostatic interactions, reducing the efficacy of peptide/membrane interaction. On the other hand, Gram-negative bacteria can acquire resistance mechanisms by inducing a positively charged lipid A, which affects the antimicrobial activity of cationic AMPs by reducing the peptide/membrane interaction. Proteolytic degradation is another mechanism in membrane-secreted proteases from bacteria, which creates a resistance concern for AMPs, a mechanism discussed previously [158,244,245,246]. As immunogenicity may also play a dual role in clinical settings, it is important to address immunogenicity-related risks in vivo before clinical trials. Immunogenicity risk assessments often involve evaluation of T-cell epitopes, which are considered key drivers of anti-drug antibody (ADA) responses and can lead to adverse effects in the host [247,248]. Although the antimicrobial activity of AMPs against MDR bacteria is evident, further optimization strategies, including AI-guided formulation and design approaches, will be required to improve their clinical translatability.

## 11. Future Directions and Outstanding Questions

The lack of AMPs’ compliance with FDA clinical trial requirements has raised major concerns and the need to involve the multi-objective optimization (MOO) field. Although MOO has emerged as a field amongst the rapid advancement of AI, its approach remains focused on laboratory-scale antimicrobial resistance and rather than addressing the AMR pandemic from a clinical standpoint. Current MOO approaches have demonstrated the creation of an accurate set of solutions, Pareto front (PF), by the “filtration” mechanisms to identify the “non-dominated” AMPs made by the Pareto ranking (PR) [249,250,251]. However, the reliance on biased human decision-making in current MOO models using a priori, a posteriori, and progressive/interactive approaches might be problematic [252]. Moreover, the PR standards for the optimal PF might need to be upgraded as FDA clinical regulatory standards are not in the picture, even though AMPs might prove effective in in vitro scenarios. Therefore, the proposal for the development of an AI/ML model with the integration of (I) hospital antibiograms, for the predictability and focus on current clinical pathogen-resistance patterns; (II) MOO capabilities, such as the PepZOO_AMP_MIC model with integrated ML/QSAR approaches, have been shown to optimize three key physicochemical properties: activity, toxicity and stability, enabling the design of target-specific AMPs; and (III) a continuous-learning algorithm to optimize the selection of the most viable AMP in the PF while the model adapts to current AMR pandemic issues [253,254]. Although current computational frameworks have not reached these capabilities in one single model yet, the integration of current effective in vitro approaches with effective clinical approaches, such as the hospital antibiograms, is a promising approach for the clinical translation of AMPs.

The rapid advancement of AI models, neural networks, and computational hardware has significantly accelerated AMP discovery and optimization. In particular, the emergence of large language models (LLMs) supports researchers in hypothesis generation, experimental design, and complex data analysis. Although LLM-based systems can predict specific protein properties, functional annotations of unknown sequences, genetic associations, disease links, and evolutionary relationships, their capacity to directly predict antimicrobial efficacy remains under active development [255]. As LLM architectures continue to evolve, their integration with structure-based modeling and quantitative predictive frameworks may enable more refined strategies for clinical AMP development. Future algorithmic platforms could assist in identifying optimal formulation and delivery approaches to enhance in vivo stability and translational potential, thereby addressing key limitations discussed in prior sections of this review, including protease susceptibility, hemolysis, cytotoxicity, solubility, and pharmacological stability. Furthermore, high-capacity pretrained protein models (e.g., ESM-1, RNG2) may contribute to multi-objective optimization pipelines, potentially serving as decision-support systems within Pareto front-guided continuous learning frameworks. As AMPs emerge as a potential strategy to address the growing AMR crisis, traditional one-size-fits-all antimicrobial approaches are unlikely to sufficiently account for the heterogeneity of intrinsic and acquired resistance mechanisms observed across MDR pathogens. For instance, among ESKAPE organisms, *E. faecium* can develop resistance to specific AMPs such as daptomycin through activation of stress-response systems, including LiaFSR, highlighting the adaptive complexity of these pathogens [256]. Rather than framing AMP therapy as “personalized,” a more clinically grounded strategy involves pathogen-directed or susceptibility-guided AMP design informed by resistance phenotypes and epidemiological data. While hospital antibiograms provide valuable localized resistance profiles, broader regional and national surveillance datasets of multidrug-resistant organisms may offer more comprehensive inputs for predictive modeling and therapeutic prioritization. In this context, emerging ML frameworks, including ML/QSAR platforms, represent promising tools for the rational design of pathogen-directed AMPs by integrating resistance data, structural descriptors, and predictive efficacy modeling. Furthermore, given the dual immunomodulatory properties of AMPs, delivery strategies such as nanoparticle encapsulation may mitigate unintended immune activation while enhancing pharmacological stability [257,258]. AI-driven models may also improve immunogenicity prediction by incorporating host-associated variables, including microbiome data, thereby supporting more precise and clinically translatable AMP development [259,260]. However, alongside these promising developments, several important limitations must be acknowledged. Predictive models are inherently constrained by the quality and heterogeneity of training datasets, which often include peptides tested under diverse and non-standardized in vitro conditions. Consequently, false-positive and false-negative predictions remain common. Moreover, antimicrobial activity demonstrated in one laboratory setting may not be reproducible under alternative experimental protocols, and translation from in vitro potency to in vivo efficacy frequently reveals additional challenges related to host interactions, protease degradation and pharmacokinetics. Importantly, most ML models explore sequence space adjacent to previously characterized peptides, raising questions about whether they enable the discovery of fundamentally novel antimicrobial scaffolds or primarily generate optimized variants of known physicochemical frameworks. Therefore, AI-guided design should be regarded as a prioritization and optimization tool that complements, rather than replaces, directed mutagenesis and targeted experimental screening strategies.

## 12. Conclusions

The currently evolving resistance mechanisms of ESKAPE pathogens and MDR fungi pose a threat to the efficacy of current antibiotics. Therefore, this problem emphasizes the urgency for researchers and clinicians to develop anti-AMR solutions to address the rapidly growing AMR pandemic. The emergence of AMPs is considered a promising solution to AMR, with demonstrated antimicrobial and antibiofilm activity against MDR pathogens. However, the in vitro and in vivo limitations discussed in this review are currently a drawback to the clinical translation of AMPs. NPs, cyclization, PEGylation, DAAs, and liposomes as delivery systems are current promising formulation strategies to overcome current limitations. Nonetheless, the extensive wet-lab procedures required to implement these formulation strategies result in high-cost screening processes of AMPs, preventing large-scale manufacturing for clinical purposes. Continuous advancements in AI-driven models are currently being developed to address the biological and manufacturing challenges that AMPs exhibit. ML and DL models are current AI-driven tools with the capability to ensure a more accurate, faster screening process, thereby lowering the cost of production in comparison with conventional wet-lab procedures. This is achieved through predictions of AMP/non-AMP classification, hemolytic activity, immunogenicity and protease degradation, therefore enhancing both manufacturing efficiency and biological safety. However, the exhibition of diverse, evolving resistance mechanisms in ESKAPE pathogens and MDR fungi discussed in this review poses a threat to current AI-driven models. Targeted antimicrobials rather than broad-spectrum antimicrobials might be a more suitable approach to address the different resistance mechanisms MDR pathogens acquire. Therefore, the development of AI-driven tools, such as continuous learning models integrating ML/QSAR algorithms and databases pertinent to clinical translation, as described in this review, is a potentially crucial approach for the design and discovery of personalized antimicrobials. The full potential of AI-driven tools has yet to unfold; however, the approach of developing LLMs into continuous learning models might be valuable for improving MOO in the design and discovery of novel AMPs, with more accurate predictions and less reliance on human-biased decision-making. As the clinical impact of AMR continues to escalate, a deeper understanding of the concepts discussed in this review will be essential to overcome current limitations in the clinical translation of antimicrobial peptides.

## Data Availability

No new data were created or analyzed in this study. Data sharing is not applicable to this article.

## Abbreviations

The following abbreviations are used in this manuscript:

AMR	Antimicrobial resistance
*C. auris*	*Candida auris*
AMPs	Antimicrobial peptides
AI	Artificial intelligence
ML	Machine learning
DL	Deep learning
MDR	Multidrug-resistant
ESKAPE	*Enterococcus faecium*, *Staphylococcus aureus*, *Klebsiella pneumoniae*, *Acinetobacter baumannii*, *Pseudomonas aeruginosa* and *Enterobacter* spp.
PJI	Periprosthetic joint infection
QS	Quorum-sensing
SVM	Support Vector Machine
RF	Random Forest
XGBoost	Extreme Gradient Boosting
CNNs	Convolutional Neural Networks
RNNs	Recurrent Neural Networks
LSTMs	Long Short-Term Memory Networks
MIC	Minimal inhibitory concentration
GANs	Generative Adversarial Networks
VAEs	Variational Autoencoders
RL	Reinforcement learning
ADMET	Absorption, Distribution, Metabolism, Excretion and Toxicity
XDR	Extensively drug-resistant
PDR	Pandrug-resistant
MRSA	Methicillin-resistant *Staphylococcus aureus*
*K.**pneumoniae*	*Klebsiella pneumoniae*
*A. baumannii*	*Acinetobacter baumannii*
*P.**aeruginosa*	*Pseudomonas aeruginosa*
*S.**aureus*	*Staphylococcus aureus*
*E. faecalis*	*Enterococcus faecalis*
*E. faecium*	*Enterococcus faecium*
AFPs	Antifungal peptides
NPs	Nanoparticles
PK	Pharmacokinetic
PEG	Polyethylene glycol
CSNPs	Chitosan-based nanoparticles
DAAs	D-amino acids
LAAs	L-amino acids
ADP	Antimicrobial Peptide Database
CAMP	Collection of Anti-Microbial Peptides
DADP	Database of Anuran Defense Peptides
DRAMP	Data Repository for Antimicrobial Peptides
DBAASP	Database of Antimicrobial Activity and Structure of Peptides
QSAR	Quantitative Structure–Activity Relationship
LSTM	Long Short-Term Memory
IV	Intravenous
PF	Pareto front
PR	Pareto ranking
MOO	Multi-objective optimization
LLMs	Large language models
ADA	Anti-drug antibodies
